# Expressive writing interventions in oncology — Reflections on expressive writing interventions in oncology

**DOI:** 10.1017/S1478951525101260

**Published:** 2025-12-29

**Authors:** Talhah Mohammed, Yasir Sakeer

**Affiliations:** King’s College London, London, UK

**Dear Editor**,

I read the scoping review on expressive writing interventions in oncology (*Palliative & Supportive Care*, 2025). The authors did a fantastic job outlining how expressive writing can enhance coping mechanisms and provide meaning to patients with cancer.

From our own reading of the literature, we also noticed similar patterns. Studies vary widely in design, participants, and outcomes. Many show improvements in emotional well-being, whereas some show little to no effect. This lends to the theory that expressive writing is not a “one-size-fits-all” approach. Its impact seemingly depends on personality types, coping styles, cultural context, and timing. We noted that expressive writing may work differently right after diagnosis, when emotions are intense, compared with once patients have had time to process their experience. More work is therefore needed to clarify when it is most effective.

Most studies follow the Pennebaker and Beall model of three or four 30-minute writing sessions. There are far fewer qualitative components, though interviews suggest that women may benefit from expressing unspoken thoughts, pointing to a gap in understanding how expressive writing actually helps.

Culture may also play a role. For example, some evidence suggests Caucasian patients benefit more from standard expressive writing than Chinese American patients. This highlights the potential value of culturally adapted approaches, though culture is just one of several factors affecting outcomes.

We also need a better understanding of why and how expressive writing works. It may help patients by changing how they think about their experience, managing emotions, or organizing their story in a way that makes sense. But in cancer populations, these ideas haven’t been studied enough. Figuring out how it works, along with when it works best and for whom, will be important for making the intervention truly effective.

Overall, expressive writing can reduce distress and restore a sense of agency and meaning. Future research should explore who benefits, how, when, and under what circumstances, using both quantitative and qualitative approaches.

Thank you

